# What kind of urban–rural basic public services can affect the urban–rural income gap?–an analysis of FsQCA based on the TOE framework

**DOI:** 10.3389/fpubh.2025.1649372

**Published:** 2025-11-06

**Authors:** Qianqian He, Tiantian Dong, Cairang Gadan

**Affiliations:** 1 School of Economics and Management, Qinghai Normal University, Xining, China; 2 Research Center for Integrated Development of Culture and Tourism, Sichuan Tourism University, Chengdu, China

**Keywords:** urban–rural basic public services, the urban–rural income gap, fsQCA, configuration analysis, TOE framework

## Abstract

**Introduction:**

The urban–rural income gap and the non-equalization of basic public services constitute the core contradiction in China’s urban–rural development.

**Methods:**

This study employs the fsQCA method based on the TOE framework to determine how technological, organizational, and environmental conditions collectively shape the urban–rural income gap in China’s Yangtze River Delta region.

**Results:**

The findings reveal three distinct configurations of high income disparity and three distinct configurations of non-high income disparity, emphasizing that no single factor is indispensable. Rather, combinations are crucial. High-disparity configurations manifest through three divergent pathways: dual squeezes from fiscal constraints and lagging digital infrastructure; structural disconnect between economic growth and digitalization; and cyclical lock-in between low-level economies and public service shortages. Non-high-disparity configurations emerge via three equivalent pathways: factor rebalancing driven by high economic output; cross-regional coordination through institutional optimization and digital empowerment; and compensatory mechanisms based on fiscal resilience and governance innovation.

**Discussion:**

The study offers recommendations for basic public service allocation across cities in China’s three major regions, holding significant implications for the integrated urban–rural development of China.

## 1 Introduction

Uneven urban–rural development is a common challenge faced by developing countries in the modernization process globally, with the deep connection between the urban–rural income gap and the uneven distribution of basic public services being particularly prominent (1). Reducing the urban–rural income gap is an important factor in promoting globalization and achieving sustainable regional development, and enhancing the efficiency of public employment services is an effective means of achieving this goal (2). In China, the dual household registration system rigidly binds access to public services to household status, creating institutional barriers for rural residents in areas such as education, healthcare, and social security (3, 4). This structural imbalance exacerbates intergenerational poverty transmission through mechanisms such as insufficient human capital accumulation and labor market segmentation (5, 6). With the continued promotion of the rural revitalization strategy, household registration system reform, and other policies, the ratio of disposable income per capita of urban and rural residents will be 2.34 in 2024, down from 2.39 in 2023, a change that reflects the trend of improvement in the pattern of income distribution in China, and the absolute gap between urban and rural incomes is continuing to narrow. However, China’s urban–rural income gap is still in the risk range of 2.0–2.5, which is at the middle to low level globally, and the problem of uneven income distribution between urban and rural areas is still relatively prominent.

The widening urban–rural income gap is essentially an economic mapping of the imbalance in the distribution of public services. Insufficient investment in early education directly constrains the rural labor force from moving to higher-skilled positions (7, 8), while the urban–rural split in social security further amplifies market risks (9). Policies in developing countries on promoting balanced regional economic development mainly support infrastructure investment and economic development in less developed regions (10).

Existing studies have mostly focused on the linear effects of single dimensions such as education investment, social security level, or industrial structure (11, 27). However, the equalization of public services is essentially a systematic project that involves the synergistic effects of technology application, organizational governance, and the external environment (12). The TOE framework was proposed by Tornatzky & Fleischer (13), and its core lies in summarizing the factors affecting organizational behaviors and technological adoption into the three types of systematic conditions–technological, organizational, and environmental–and emphasizing the interaction mechanism among the three. The framework integrates the diffusion of innovation theory and the technology acceptance model (14) and has been widely used in the fields of technology adoption, digital transformation, and other management research because it can analyze complex organizational phenomena from a multilevel dimension (15, 16).

The practice of cross-regional governance innovation and digital technology application in the Yangtze River Delta region provides a typical scenario for exploring the group effect of multiple conditions. Traditional measurement methods are limited by linear assumptions, making it difficult to capture “multiple concurrent causal” relationships, while fuzzy set qualitative comparative analysis (fsQCA) is able to identify the combinatorial effects of conditioned variables and reveal asymmetric causal pathways (17, 18), which is highly compatible with the TOE framework’s emphasis on the interaction of technological, organizational, and environmental conditions (19). Therefore, combining the TOE framework with fsQCA helps to systematically analyze the synergistic or substitution effects of multiple factors in the allocation of basic public services in urban and rural areas and breaks through the limitations of traditional single-factor analysis.

Taking the Yangtze River Delta (YRD) urban agglomeration as the study area, this study utilizes the fsQCA methodology to explore the complex association between urban–rural basic public service allocation and income disparity based on the TOE framework, with the aim of answering the following questions: First, which core elements of urban–rural basic public services have a significant impact on urban–rural income disparity? Second, are there multiple paths of urban–rural basic public service combinations that can affect the urban–rural income gap? The urban agglomeration of the Yangtze River Delta (YRD) combines a gradient difference in economic development with an early advantage in institutional innovation, which makes the YRD cities an ideal region to study the configuration effects of the urban–rural gap, not only to verify the “trickle-down effect” of the external environment on the income gap, but also to examine the substitution mechanism of the financial constraints imposed by the organizational and technological conditions (20).

This study breaks through the traditional single-factor analysis framework and reveals the “group dependence” of the urban–rural income gap from the perspective of the TOE framework, aiming at proving that the balanced development of urban and rural areas does not only depend on the strength of a single policy instrument but also depends on the synergistic or substitution effects of technical, organizational, and environmental conditions. By placing urban–rural basic public services in the TOE framework for group analysis, this study provides a new theoretical perspective for urban–rural income disparity research, which not only enriches the multidimensional analytical framework for urban–rural disparity, research but also provides “group optimization” ideas for the deepening of the integration strategy of the Yangtze River Delta.

## 2 Literature review and theoretical background

### 2.1 Basic public services in urban and rural areas

As a core component of social policy, urban and rural basic public services refer to government-led provision of basic services directly related to the right to survival and development of urban and rural residents, covering such areas as education, health care, social security, infrastructure, public health, etc. (4, 21). Its essence is to guarantee the basic rights of citizens through its organizational conditions; however, in developing countries affected by the dual economic structure and institutional barriers, the non-equalization of basic public services in urban and rural areas has become a prominent problem (1).

In China, the distribution of basic public services between urban and rural areas is constrained by institutional factors. It links public identity to access to public services, forming a “rural–urban” dichotomy (22). This manifests in rural residents being excluded from urban public service systems even after prolonged urban employment. Rural migrant workers can only enroll in the New Rural Cooperative Medical Scheme (NRCCM) at their place of residence, where hospitalization reimbursement rates are significantly lower than those under the basic medical insurance for urban employees (23).

Under the TOE framework perspective, the essence of urban–rural basic public services is a comprehensive reflection of organizational, environmental, and technological conditions. Government governance needs to integrate various tools such as finance, land, and industry, and the non-equalization of urban and rural public services in China exposes the dual deficiencies of governance capacity and financial capacity (12). Existing research also reveals that there are significant differences in the “soft” and “hard” structures of public services. “Soft” public services (education, healthcare, social security) have a much higher moderating effect on income disparity than “hard” public services (infrastructure) (24). Differential investment in early education has the highest contribution to income disparity in China, and subsidizing early education is seen as the most effective policy (8), while insufficient investment in education in rural areas directly constrains the accumulation of human capital and creates a cycle of intergenerational poverty (5, 6).

In summary, the disparity in basic public services between urban and rural areas stems from the combined effects of government governance, economic development, and technological innovation. Addressing this challenge requires focusing on organizational conditions within the TOE framework, such as optimizing fiscal transfer mechanisms and enhancing local governments’ collaborative governance capabilities (20, 25). These theoretical studies provide a research framework for analyzing urban–rural income disparities and lay the theoretical foundation for this study’s exploration of pathways for public service grouping.

### 2.2 Urban–rural income gap

The urban–rural income gap is a concentrated manifestation of structural contradictions in developing countries. In China, this gap shows typical stage characteristics, and although it has declined after policy interventions such as rural revitalization, the absolute gap still persists along with the non-equalization of public services (26). The perpetuation of the gap not only inhibits the expansion of the consumer market but also creates a “poverty cycle” through the intergenerational distribution of resources such as education and healthcare, and the lack of investment in the human capital of rural families has led to barriers to the career advancement of their children, exacerbating the solidification of the social stratification (5, 6).

Institutional constraints form the core explanatory factor for the urban–rural income gap. By restricting access to public services, they result in inadequate coverage of pension insurance for migrant workers, forcing them to enroll in the New Rural Cooperative Medical Scheme only in their registered hometowns (9). The double-edged sword effect of economic structural transformation is also pronounced: industrial upgrading in eastern regions widens the gap initially through an “N-curve” before narrowing it, while western regions experience an expanding gap due to labor skill mismatches (27). Urbanization, while widening disparities in the short term through factor concentration, converges gaps in the long run via the spillover effects of public services, thereby constraining human capital development (28).

Social capital differences amplify income differentiation through the human capital pathway. Unequal distribution of educational resources contributes 35% to the income gap, with insufficient investment in early education directly constraining rural labor mobility to higher-skilled jobs (7, 8), and urban–rural disparities in healthcare resources reduce labor productivity through health human capital depletion (29, 30), as evidenced by the fact that urban households are generally better equipped with soap and water hand-washing facilities than rural households (31). Under the perspective of the TOE framework, the misconfiguration of technological, organizational, and environmental conditions exacerbates the structural contradictions, and most of the existing research focuses on the linear effects of single factors and seldom studies the group relationship among variables of each dimension. Based on the TOE framework, this study introduces fuzzy set qualitative comparative analysis (fsQCA), aiming to reveal the multiple concurrent causal relationships of the condition variables, break through the explanatory limitations of the traditional linear model, and provide a new theoretical perspective for the systematic optimization of urban–rural integration policies.

### 2.3 Impact of urban–rural basic public services on the urban–rural income gap

The impact of urban–rural basic public services on the urban–rural income gap is a core topic of development economics and institutional research, and its mechanism runs through the multiple dimensions of resource allocation, human capital accumulation, and government governance. As a core public service area, the distribution of educational resources is an important factor affecting the urban–rural income gap. Becker & Chiswick (32) were the first to reveal the decisive role of education differences in income differentiation. Yang & Qiu (8) further found that differences in China’s investment in early childhood education contributed 35% to the income gap and proposed that the subsidization of rural preschool education is the most effective policy tool. The rural–urban split in social security, on the other hand, exacerbates imbalances through labor market discrimination, with insufficient levels of social security in rural areas directly widening the income gap (33), while research in India suggests that the lack of formal insurance leads to labor mismatches and the formation of a spatial wage divide (34).

The impact of “hard” public services such as infrastructure varies regionally. For instance, China’s high-speed rail construction has shown a weak convergence effect on urban–rural income disparities (35). However, in underdeveloped regions, low internet penetration rates (IPR) may create a “digital poverty trap” hindering rural residents’ access to emerging employment opportunities like e-commerce (36). A study employing triadic graph analysis found that disparities in basic healthcare service levels between urban and rural areas are highly correlated with residential space. Across 73 countries, basic healthcare coverage in urban areas significantly exceeds that in rural areas, suggesting that unequal distribution of public service resources may exacerbate urban–rural income gaps through human capital pathways (31). However, institutional constraints weaken the poverty-reducing effects of the former (24). Disparities in educational investment reinforce income stratification through the intergenerational transmission of human capital (6). Inadequate coordination between policy design and governance capacity exacerbates structural contradictions; achieving equitable public services requires integrating multidimensional policy instruments across finance, land, and industry (20). Regions with significant income disparities often lag in basic public services and social governance, leading to economic imbalances (25).

Established studies on the impact of urban–rural basic public services on the urban–rural income gap mostly focus on the linear impact of a single factor and seldom explore the group effects among the dimensions of public services from the perspective of the TOE framework. Based on the TOE framework, this study introduces fuzzy set qualitative comparative analysis (fsQCA), aiming to reveal how conditional variables affect the urban–rural income gap through synergistic or substitution effects and to provide a nonlinear explanatory framework and policy recommendations for solving the urban–rural basic public service allocation challenges.

### 2.4 The TOE framework

The TOE framework was proposed by Tornatzky & Fleischer (13), which centers on summarizing the factors affecting organizational behavior and technology adoption into three types of systemic conditions, namely, technology, organization, and environment, and emphasizes the interaction mechanism among the three. The framework integrates the diffusion of innovation theory and technology acceptance model (14) and has been widely used in management research fields such as technology adoption and digital transformation because it can analyze complex organizational phenomena from multilevel dimensions (15, 16). In industries such as healthcare and e-commerce, scholars have used the framework to identify the differential impacts of technological capabilities, organizational resources, and external market environment on technology adoption (37, 38). Its theoretical strength lies in capturing the synergistic logic of technology-organization-environment through a systemic perspective (39), providing a structured analytical framework for analyzing multifactor interactions.

In the technology dimension, the TOE framework focuses on the technological status of organizations, covering descriptive indicators such as technological capabilities, government pressure, and resources (40). For example, in the context of Industry 4.0, digital technologies such as artificial intelligence and big data governance have become key enablers of entrepreneurial activities by reshaping the global value chain (41, 42), suggesting that technological sophistication drives organizational effectiveness (43). Organizational dimensions focus on internal attributes, including resource availability, organizational rules, degree of technological need, and strategic orientation (44, 45). Organizational absorptive capacity for technology, top management support, and human resource reserves directly affect the depth and breadth of technology adoption (46, 47). The environmental dimension involves the organization’s external ecosystem, which contains market competition, policies and regulations, and industry characteristics (48). For example, a favorable business environment (e.g., low entry costs, land availability) significantly promotes SME growth (49), whereas government pressure and industry competition patterns constitute external constraints on technology adoption (40).

The TOE framework provides researchers with a strong theoretical foundation and explains the influence of many factor groups on the technological activities of firms (48), and thus the integration of the TOE framework with fuzzy-set qualitative comparative analysis (fsQCA) provides a unique advantage in the analysis of groupings of complex social phenomena. fsQCA’s nonlinear causal logic is highly compatible with the TOE framework’s The concept of interaction of technological, organizational, and environmental conditions is highly compatible (18, 19) and is able to identify synergistic paths of multiple conditions through multi-case comparisons. In the research scenario of urban–rural basic public services and income disparity, this combination can break through the limitations of traditional single-factor analysis and systematically analyze the configuration effect in the TOE framework. Through the configuration analysis of fsQCA, this study aims to reveal the key combinations of conditions affecting the urban–rural income gap and to provide an empirically based analytical paradigm and theoretical basis for the expansion of the application of the TOE framework in urban–rural basic public services.

## 3 Research methods and data

### 3.1 Methods of analysis

#### 3.1.1 Qualitative comparative analysis of fuzzy sets and necessary condition analysis

This paper incorporates both Necessary Condition Analysis (NCA) and Qualitative Comparative Analysis (QCA) research methods, focusing on exploring two types of causal associations: first, exploring whether a particular element constitutes a necessary condition for city district to achieve a low urban–rural income gap and the extent to which the element plays such a role; and, second, analyzing how these elements interact and combine to contribute to a high urban–rural income gap or non-high urban–rural income disparities.

Qualitative Comparative Analysis (QCA) is a set-theoretic approach using Boolean algebra principles to identify different combinations of attributes that lead to necessary or sufficient conditions for a particular outcome (18). Fuzzy-set qualitative comparative analysis (fsQCA) is particularly good at revealing different groupings of sufficient and/or necessary conditions that lead to a relevant outcome (50). The fuzzy-set qualitative comparative analysis (fsQCA) method skillfully blends the strengths of qualitative and quantitative analyses and, to a certain extent, fills the gap between large-sample quantitative analyses in terms of capturing qualitative variations and the depth of complex phenomena. The method not only enhances the general applicability and explanatory power of the research results but also ensures the meticulousness of the analysis process. Compared with traditional regression analysis methods, the core of the fsQCA method lies in the use of Boolean algebraic logic, a feature that fundamentally avoids the problem of omitted variable bias. During fsQCA analysis, the researcher does not need to set control variables, thus allowing more freedom to explore how multiple factors work together to contribute to an outcome (51).

This paper employs the Necessary Condition Analysis (NCA) method (17) and effectively combines the QCA method, which has excelled in the analysis of sufficient conditions, in particular the fuzzy-set qualitative comparative analysis (fsQCA). Although fsQCA is capable of identifying necessary conditions, its formulation is limited to a qualitative level and fails to quantitatively demonstrate the extent of necessary conditions. NCA, on the other hand, can provide a complement in this regard, especially when dealing with fuzzy sets, and its rich affiliation scores make the use of NCA in combination with fsQCA even more valuable in revealing complex causality in a more comprehensive manner (52).

### 3.2 Research model

**Figure 1:**
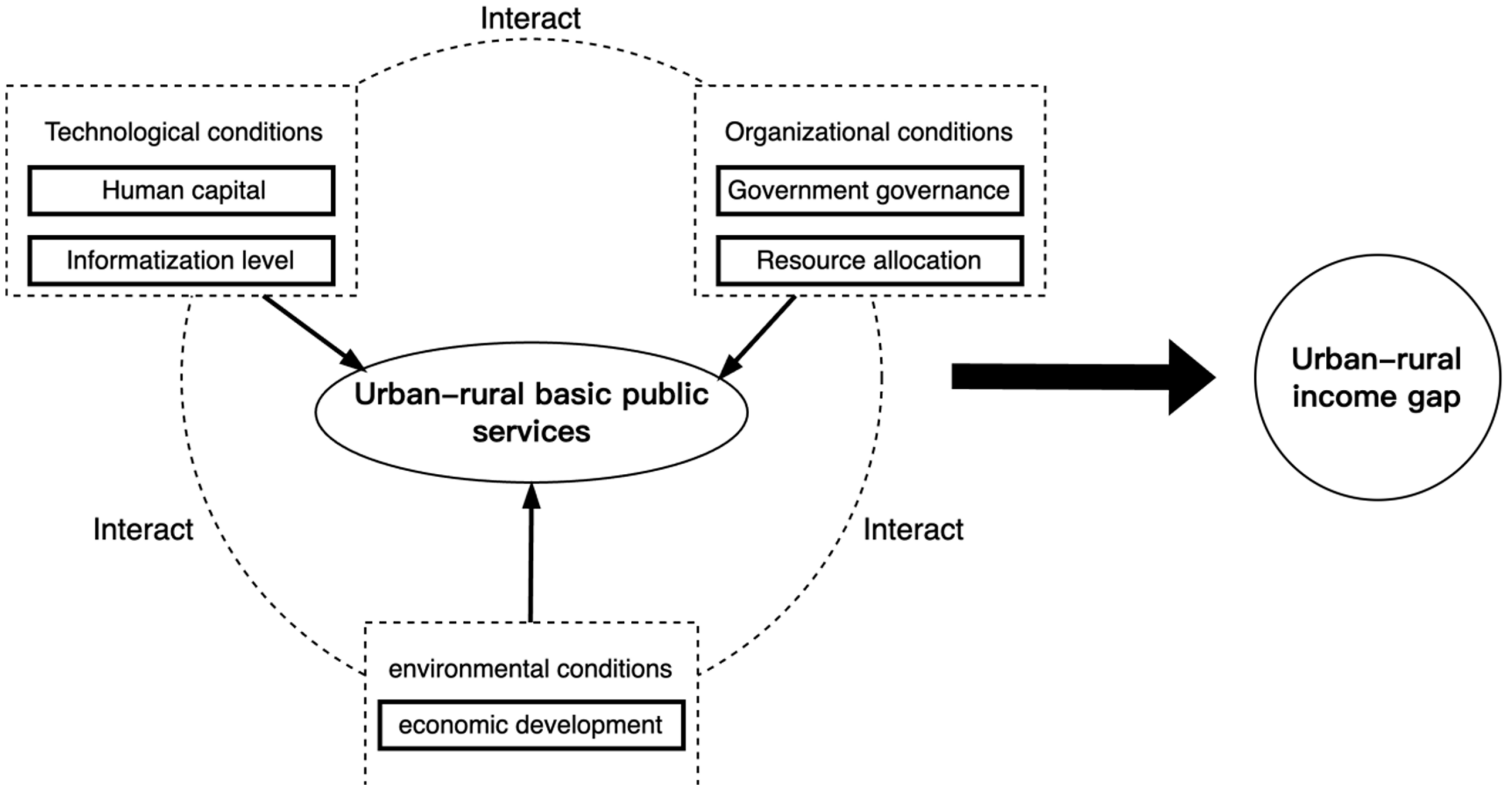
Analytical model.

This study builds a theoretical model based on the TOE framework and constructs conditional variables of urban–rural basic public services based on technological, organizational, and environmental conditions: technological conditions cover human capital and informationization level, organizational conditions focus on government governance and resource allocation, and environmental conditions focus on the level of economic development. Through the interaction effect among the three types of conditions, the degree of equalization of urban–rural basic public services is presented so as to analyze the complex causal mechanism of the urban–rural income gap under the multi-conditional grouping. Therefore, this study proposes the following theoretical model (Figure 1) for subsequent analysis using the fsQCA 4.1 software (Figure 1).

### 3.3 Data sources

The data for this study are derived from the 2023 China Statistical Yearbook, provincial and municipal statistical yearbooks or bulletins, the GuotaiAn Regional Economic Database, the China Urban Statistical Yearbook, and the Evaluation Report on Political-Business Relationships in Chinese Cities.

### 3.4 Measurement of variables

Technological, organizational, and environmental conditions are taken as the measurement dimensions of the condition variables, and the subdivided variables under each dimension are intergovernmental relations, government-market relations, government-society relations, economic development, human capital and informatization level, as shown in Table 1.

**Table 1:** Summary of the variables used in the model.

Variable type	Dimension of measurement	Segmentation variables	Measurement indicators
outcome variable		Urban–rural income gap	Tyrell’s index
conditional variable	Organizational conditions	Intergovernmental relations	Fiscal self-sufficiency rate
		Government-market relations	Political-Business Relationship Index
			Government cleanliness index
		Government-society relations	Public service expenditures
	Environmental conditions	Economic development	Per capita GDP
	Technological conditions	Human capital	Number of full-time teachers in general higher education
		informatization level	Internet penetration

#### 3.4.1 The Theil index

The Thiel index is used as an outcome variable to assess the overall amount of urban–rural income discrepancy. This indicator is calculated by comparing the proportion of urban and rural residents’ incomes to the proportion of the population, and it has the advantage of more accurately reflecting the income disparity between urban and rural population shares, with larger values indicating higher levels of inequality. The formula for measuring the Theil index is as follows (Equation 1):


$${\text{Theil}}_{t}=\frac{Y_{1t}}{Y_{t}}\text{In}(\frac{Y_{1t}/P_{1t}}{Y_{t}/P_{t}})+\frac{Y_{2t}}{Y_{t}}\text{In}(\frac{Y_{2t}/P_{2t}}{Y_{t}/P_{t}})$$ (1)

Where $Y_{1t}$ and$\;Y_{2t}$ denote the total income of urban and rural residents in period t, respectively (obtained by multiplying the total population and the per capita income level);$\;Y_{t}$ is the sum of the total incomes of urban and rural residents in period t;$\;P_{1t}$ and$\;P_{2t}$ denote the population size of urban and rural areas in period t, respectively; and$\;P_{t}$ is the total population size in period t.

#### 3.4.2 Technological condition

The technology dimension focuses on the technological status and innovation capacity of organizations, including human capital and digitalization level (41, 42). Endogenous growth theory states that human capital accumulation is the core driver of technological progress, which drives sustained economic growth through knowledge creation and spillover effects. Differences in early education investment have the highest contribution rate to the income gap in China (8), and in this study, the number of college students per 10,000 students commonly used as an indicator for measuring human capital in Chinese cities is selected to represent the level of human capital, reflecting regional knowledge reserves and innovation potential (43), which is a key element in promoting the technologization and precision of public services in urban and rural areas; the Internet penetration rate, as a measure of the level of informatization, reflects the degree of penetration of digital technology in urban and rural society, and is closely related to the application of technologies such as big data governance and intelligent service platforms in the context of Industry 4.0 (41). Endogenous growth theory emphasizes the facilitating effect of technological diffusion on knowledge spillover, and digital technology can break through the geographic boundaries of traditional public services and enhance the accessibility of services in rural areas through the sharing of educational resources, medical remote diagnosis, etc. (42), whereas the accumulation of human capital strengthens the effectiveness of the application of technology, and the two together constitute the technological configuration of urban and rural public service allocation. The two together constitute the technological support for the allocation of urban and rural public services, echoing the theoretical kernel of endogenous growth theory in which technology and human capital synergistically drive development.

#### 3.4.3 Organizational conditions

The TOE framework of organizational conditions focuses on the role of intra-organizational attributes on technology application and decision-making, covering core elements such as resource availability, organizational rules, and policy support (44, 45). According to public choice theory, this study refines organizational conditions into three types of indicators: intergovernmental relations, government-market relations, and government-society relations. Intergovernmental relations are measured by the financial self-sufficiency rate, which not only reflects the financial autonomy of the local government but also fits the assumption of “bureaucratic budget maximization” in public choice theory and reveals that resource allocation barriers are formed by financial competition between governments. The government-market relationship is measured by the government-business relationship index and the government cleanliness index, which correspond to the public choice theory’s criticism of rent-seeking behavior and quantitatively assess the risk of resource mismatch due to collusion or corruption among interest groups; the government-society relationship is measured by the public service expenditure index, which echoes the “middle-voter theorem,” reflecting the problem of inadequate rural service provision and inefficient resource allocation due to voter preference bias in government spending decisions (45). Resource support at the organizational level is the foundation of technology application and social governance (53), and the improvement of governmental governance capacity and rational allocation of resources can alleviate income inequality by narrowing the urban–rural public service gap.

#### 3.4.4 Environmental conditions

Environmental conditions focus on the organization’s external ecosystem, including market competition, policy environment, and level of economic development (49, 54). From the perspective of growth pole theory, the economic development conditions of a particular region are a reflection of the “agglomeration effect” of economic growth poles, which in turn indicates that balanced economic development has a significant impact on the equalization of basic public services between urban and rural areas. This study measures economic development by GDP per capita, which not only reflects the overall scale of regional wealth but also implies the differences between urban and rural industrial structure and factor flow patterns. From the perspective of environmental embeddedness, regions with higher levels of economic development usually have more abundant financial resources for public service provision, while the process of industrialization and urbanization may accelerate urban–rural population mobility, forcing the optimization of the public service system (49). The imbalance of economic development may also lead to the agglomeration of resources to cities, exacerbating the urban–rural income gap (54). Therefore, the level of economic development is not only the material basis for urban and rural basic public service inputs but also an environmental constraint that affects the effectiveness of technology application and the efficiency of policy implementation, which needs to be examined in the group analysis of its interaction with technological and organizational conditions.

### 3.5 Data calibration

In this study, the observed variables are calibrated using the direct method, which involves changing them into fuzzy-sets. Based on the treatment in the published literature (55), “three anchor points” for the four condition variables and one outcome variable (Thiel index) are selected: “full membership,” “crossover point,” and “full non-membership.” Specifically, the anchor points for the sample data are the upper quartile (75%), median, and lower quartile (25%). The calibration of the non-high urban–rural income gap is achieved by excluding the high urban–rural income difference. Table 2 shows the specific calibration findings for each study variable.

**Table 2:** Data calibration.

Variable classification	Variable description		Full membership	Crossover point	Full non-membership
outcome variable	TI	Urban–rural income gap	0.0617	0.0394	0.0266
conditional variable	FSR	Fiscal self-sufficiency rate	0.6588	0.5167	0.4119
	GBRI	Political-Business Relationship Index	64.5	47.32	41.82
	GII	Government cleanliness index	67.08	49.09	43.63
	PGDP	per capita GDP	147,057	93,731	76,853
	PSE	Public service expenditures	89.87	61.87	34.53
	HC	The ratio of college students to the total local population	297.87	215.26	128.26
	IPR	Internet penetration rate	6.5206	4.6107	3.9134

## 4 Conclusion

### 4.1 Necessity analysis

This study empirically examines the possible adequacy or necessity of the relationship between conditional variables and outcome variables in terms of the dimensions of consistency and coverage. Using the fsQCA 4.1 analysis tool, reference was made to established research (56), and in order to ensure the reliability of the study’s conclusions, a systematic analysis was also carried out to analyze the situations where the condition variable had a missing status (marked with a “~“). The empirical results show (Table 3 for details) that the consistency of individual conditional necessities is below the threshold of 0.9 for determining the necessity condition, a finding that suggests that, in the case of urban–rural basic public services, none of its dimensional indicators meets the criteria of the necessary preconditions that drive the urban–rural income gap.

**Table 3:** Results of necessity analysis for individual condition variables.

Conditional variable	Description of variables	High urban–rural income gap		Non-high urban–rural income gap	
		Consistency	Coverage	Consistency	Coverage
FSR	High financial self-sufficiency	0.328880	0.326864	0.730269	0.803371
~FSR	Low financial self-sufficiency	0.802158	0.728758	0.388115	0.390289
GBRI	High political and business relations index	0.320658	0.307541	0.740018	0.785609
~GBRI	Low Political-Business Relationship Index	0.776465	0.729599	0.347725	0.361661
GII	High government cleanliness index	0.449126	0.450283	0.587279	0.651726
~GII	Low government innocence index	0.652621	0.588235	0.504642	0.503474
PGDP	High GDP per capita	0.285200	0.268765	0.811049	0.846005
~PGDP	Low GDP per capita	0.836588	0.800000	0.298979	0.316462
PSE	High public service expenditures	0.313464	0.295400	0.783194	0.816949
~PSE	Low public service expenditures	0.805755	0.770516	0.324513	0.343489
HC	High ratio of university students to total local population	0.292909	0.277778	0.800371	0.840156
~HC	Low ratio of university students to total local population	0.831449	0.790039	0.311978	0.328125
IPR	High Internet penetration	0.316547	0.300488	0.804550	0.845366
~IPR	Low Internet penetration	0.837102	0.794634	0.334262	0.351220

### 4.2 Pathways and case studies

In this study, the consistency threshold is set at 0.8, and the case cutoff value is set at 2. Based on the uncertainty of the direction of the influence of environmental conditions, the symmetry assumption is used to conduct a counterfactual analysis by comparing the intermediate solution with the parsimonious solution to identify the core conditions and the edge conditions (55). The histogram analysis paths are shown in Table 4, with several driving paths (H1-H6) for high urban–rural income gap and several inhibiting paths (L1-L7) for non-high urban–rural income gap. The results of the group analysis are shown in Table 5, with total coverage of 0.685 and 0.736, which can explain 68.5 and 73.6% of the sample cases, respectively, and the overall consistency is higher than 0.9, which verifies the robustness of the analysis results. Each grouping that affects the urban–rural income gap is analyzed in detail below.

**Table 4:** Results of the configuration analysis.

Solutions	High urban–rural income gap	Non-high rural–urban income gap
Parsimonious solution	~PSE* ~ IPR ~FSR* ~ PGDP	PSE FSR*IPR
Complex (intermediate) solution	~GBRI* ~ PGDP* ~ PSE* ~ HC* ~ IPR ~FSR* ~ GBRI* ~ GII* ~ PGDP* ~ PSE* ~ IPR ~FSR* ~ GBRI* ~ GII* ~ PGDP* ~ PSE* ~ HC ~FSR*GII* ~ PGDP* ~ PSE* ~ HC* ~ IPR FSR* ~ GBRI*GII* ~ PSE* ~ HC* ~ IPR ~FSR*GBRI*GII* ~ PGDP*PSE*HC* ~ IPR	FSR*GBRI*PGDP*HC*IPR FSR*GBRI*GII*PSE* ~ HC*IPR FSR* ~ GII*PGDP*PSE*HC*IPR GBRI* ~ GII*PGDP*PSE*HC*IPR ~FSR* ~ GBRI* ~ GII*PGDP*PSE*HC* ~ IPR FSR* ~ GBRI* ~ GII*PGDP* ~ PSE* ~ HC*IPR ~FSR*GBRI*GII* ~ PGDP*PSE*HC* ~ IPR

*Means Boolean logic “and”; ~ means Boolean logic “not”.

**Table 5:** Paths of high and non-high urban–rural income gap.

Conditional variable	High urban–rural income gap						Non-high urban–rural income gap						
	H_1a_	H_1b_	H_2a_	H_2b_	H_3a_	H_3b_	L_1a_	L_1b_	L_2a_	L_2b_	L_3a_	L_3b_	L_3c_
FSR		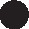	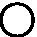	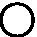	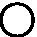	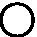	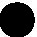	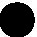	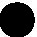	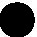	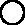		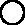
GBRI	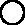	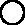	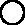		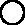	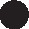	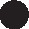	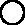		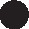	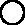	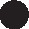	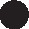
GII		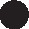	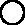	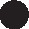	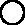	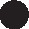		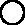	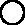	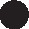	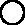	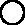	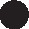
PGDP	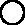		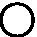	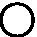	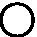	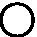	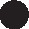	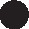	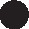		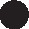	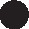	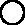
PSE	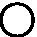	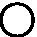	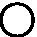	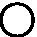	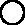	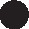		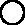	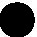	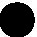	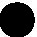	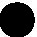	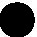
HC	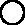	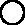		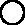	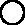	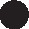	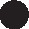	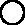	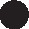	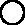	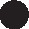	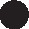	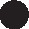
IPR	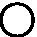	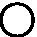	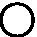	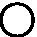		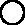	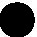	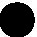	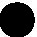	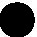	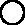	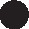	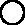
Solution coverage	0.684995						0.73584						
Solution consistency	0.955556						0.931805						

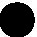
 indicates that the core condition exists; 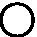 indicates that the core condition is missing; 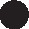 indicates that the marginal condition exists; 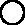 indicates that the marginal condition is missing; and a blank space indicates that the condition may or may not exist.

#### 4.2.1 Analysis of the high urban–rural income gap

##### 4.2.1.1 Inadequate public services and digital infrastructure path (H_1a_, H_1b_)

The core missing conditions of this path are insufficient public service spending and low Internet penetration, accompanied by marginal missing conditions such as poor government-business relations and weak human capital, with typical cases such as Xuzhou in northern Jiangsu. Xuzhou’s rural areas face a serious imbalance in public service investment over time, with per capita public service expenditure in rural areas only 36.7% of the urban level in 2022; the Internet penetration rate in rural areas is 52.6% in 2023, and digital and e-commerce infrastructure is lagging behind, making it difficult for education and healthcare resources to cover the rural areas efficiently and effectively; meanwhile, low government-business coordination efficiency and the proportion of skilled labor further exacerbate the low allocation of resources. At the same time, the low efficiency of government-business coordination and the proportion of skilled labor further exacerbate the inefficiency and mismatch of resource allocation, hindering the transformation of traditional agriculture into a digital distribution system. It is recommended to establish a cross-regional coordination mechanism for the provision of public services in the Yangtze River Delta, increase targeted investment in rural digital infrastructure, and simultaneously promote the standardization of government-business relations and the construction of vocational skills training systems.

##### 4.2.1.2 The path of total factor resource scarcity (H_2a_, H_2b_)

This path is characterized by multiple deficiencies in core conditions such as financial autonomy, per capita GDP, public service expenditure, and Internet penetration, as in Chuzhou, Anhui Province. This type of city is limited by weak fiscal autonomy, low economic aggregate, rural public service provision, and digital technology application caught in systematic deficiencies. 2023 Chuzhou rural public service expenditure accounted for only 28.1%, the Internet penetration rate was 38.5%, the traditional industrial structure constrained on the fiscal blood-creation capacity, the formation of lagging economic development, insufficient fiscal drawing capacity, shortage of public services, and digital penetration of the weak composite dilemma. It is necessary to upgrade the economic capacity through the Yangtze River Delta Industrial Cooperation Park, set up a special fund for fiscal transfer payments and digital infrastructure, and clarify the rigid proportion of rural public service expenditures.

##### 4.2.1.3 The path of fiscal and economic recession accompanied by institutional manpower shortage (H_3a_, H_3b_)

This path is characterized by the absence of core conditions such as financial self-sufficiency rate and GDP per capita and the absence of marginal conditions such as political and business relations index and human capital, typical of Huainan in northern Anhui Province. Because of the long-term dependence on resource-based industries, the fiscal self-sufficiency rate is below the critical value and the economic structure is single, which is compounded by the unstandardized government-business relationship and the loss of human capital, and the supply of rural public services lacks sustainable support. Huainan’s rural labor force lacks professional skills, and the allocation of public service resources is inefficient. It is necessary to promote the transformation of resource-based industries into high value-added sectors to enhance financial resilience, introduce the experience of the core cities of the Yangtze River Delta in the governance of government-business relations, and establish incentives for the return of talent and a platform for cross-regional cooperation in vocational education.

#### 4.2.2 Analysis of the non-high urban–rural income gap

##### 4.2.2.1 Fiscal capacity digital technology synergy path (L_1a_, L_1b_)

This path is characterized by the existence of core conditions such as fiscal self-sufficiency and Internet penetration rate, and marginal conditions such as GDP per capita, typical of Shanghai Qingpu. Typical examples include Qingpu, Shanghai. This type of city relies on high financial self-sufficiency and Internet penetration to form the foundation of urban–rural integration with financial support and digital empowerment. Through the construction of the Yangtze River Delta Eco-Green Integrated Development Demonstration Zone, Qingpu has utilized the advantage of financial resources to promote the full coverage of rural 5G networks, with the coverage rate of the rural digital government platform reaching 92% in 2024 and more than 300 items of government services being “cross-provincial,” effectively breaking down administrative barriers. The existence of per capita GDP edge conditions has strengthened the absorption capacity of industrial upgrading for rural labor, and local rural residents are involved in e-commerce operations, smart agriculture, and other new businesses through digital skills training, and the proportion of non-farm income has increased significantly. Policies need to deepen the cross-regional financial synergy mechanism, incorporate digital infrastructure standards into the framework of urban–rural public service integration, and promote the deep penetration of digital technology into rural livelihoods.

##### 4.2.2.2 Fiscal digital service synergistic promotion path (L_2a_, L_2b_)

The path is manifested by the existence of the fiscal self-sufficiency rate, the Internet penetration rate, and the core conditions of public service expenditure, with typical cases such as Suzhou. Suzhou, with high financial self-sufficiency to support public service spending to rural tilt, 2023 urban and rural residents of the basic health insurance financial subsidy standard unification of 1,200 yuan per person per year, relying on high Internet penetration to build a rural e-commerce ecosystem, Kunshan and other places in the rural e-commerce turnover growth rate of more than 25% per annum. The group states that through financial protection, equal service, and digital efficiency synergistic mechanisms, it will realize the balanced allocation of urban and rural public service resources and rural economic industry upgrading. It is recommended to further optimize the structure of fiscal expenditure, incorporate the digital transformation of public services into the budget assessment, and establish precise supply mechanisms such as “digital service vouchers” to enhance the accessibility of digital public services for rural residents.

##### 4.2.2.3 Public service-led human capital support path (L_3a_, L_3b_, L_3c_)

This path is characterized by the existence of core conditions for public service expenditure and marginal conditions for human capital levels, with typical cases such as Changzhou. Changzhou promotes the balanced allocation of urban and rural education and medical resources through high public service expenditures, and in 2023, the standard of per-pupil public funding for compulsory education in rural areas will be the same as that in urban areas, and the equipment allocation of primary medical institutions will reach the standard rate of 95%. The existence of marginal conditions for the level of human capital provides support for the transformation of the effectiveness of public services, relying on the resources of local universities to carry out “new vocational farmers” training, annual training of rural e-commerce talents, and the formation of a virtuous cycle of service provision, manpower enhancement, and income growth. Policies need to strengthen the linkage mechanism between public services and human capital cultivation, such as the establishment of a special fund for rural education and health care and the simultaneous establishment of a “human capital points” system that links skills training to eligibility for public services, so as to enhance the efficiency of resource allocation.

### 4.3 Sensitivity analysis

This study conducted robustness tests on the pre-configurations of high urban–rural income disparities and non-high urban–rural income disparities (57). In the fsQCA4.1 software, the consistency threshold for configurations generating high and non-high urban–rural income disparities was raised from 0.8 to 0.85, yielding configurations largely consistent with existing results.

Robustness tests revealed no substantial deviation in configuration paths, maintaining a clear subset relationship with the original solution, indicating stable findings.

Notably, the stricter parameter threshold essentially eliminated peripheral solutions while preserving core configurations that satisfy enhanced logical rigor (18). This strategic refinement confirms the persistence of core causal configurations under stricter analysis. Based on these tests, the study’s findings demonstrate robustness.

## 5 Discussion and implications

### 5.1 Main findings

Based on the TOE framework, this study uses fuzzy set qualitative comparative analysis (fsQCA) to explore the group relationship between urban–rural basic public service allocation and urban–rural income disparity in the Yangtze River Delta (YRD) urban agglomeration and finds that both the high urban–rural income disparity and the non-high urban–rural income disparity are caused by multifactorial interactions, and there is a differentiated driving path.

The high urban–rural income gap is driven by three types of groupings: first, the path of insufficient public services and digital infrastructure, characterized by a core lack of public service expenditures and Internet penetration, and the superimposition of marginal conditions such as poor government-business relations and weak human capital; second, the path of scarcity of total factor resources, which presents a lack of total factors such as the fiscal self-sufficiency rate, per capita GDP, and public service expenditures; and, third, the path of fiscal and economic decline accompanied by a lack of human capacity in the system. The third is the path of fiscal and economic recession accompanied by institutional manpower shortage, characterized by the core lack of fiscal self-sufficiency rate and GDP per capita, accompanied by the irregularity of government-business relations and the loss of human capital.

Three types of paths exist for non-high urban–rural income gaps: digital technology synergy of financial capacity, synergistic promotion of financial digital services, and public service-led human capital support. The study shows that single conditions are not necessary for the urban–rural income gap and that the grouping effects of technological, organizational, and environmental conditions are key, providing a non-linear explanatory framework for urban–rural integration policies.

### 5.2 Theoretical implications

This study combines the TOE framework with the fsQCA method, breaks through the theoretical limitations of traditional linear causal logic, and provides a new theoretical perspective of “group dependence” for the study of the urban–rural income gap. Existing literature mostly focuses on the influence of single factors such as education investment, city size, and social security level on the urban–rural income gap but seldom explores the interaction mechanism of technology, organization, and environmental conditions. This study confirms that the formation of the urban–rural income gap is not the result of a single condition but the product of the synergy or substitution of multiple groupings of technological, organizational, and environmental conditions, which expands the boundary of the application of the TOE framework in the field of public services and provides a theoretical paradigm for the understanding of non-linear causality of complex social phenomena.

At the methodological level, the study identifies the differentiated driving paths of the high urban–rural income gap and the non-high urban–rural income gap through the fsQCA method, revealing the characteristic of “multiple concurrent causation,” and fills the gap in the traditional measurement method in terms of capturing the conditional combination effect of urban–rural basic public services. This research paradigm, which combines the TOE framework with group analysis, not only enriches the multidimensional analytical framework of urban–rural disparity research but also provides methodological innovations for the study of complex issues in the field of public services.

### 5.3 Policy and administrative implications

The study provides systematic governance ideas for cracking the urban–rural dichotomy: in the technological dimension, accelerating the penetration of digital technology and talent cultivation in rural areas, and breaking through geographic boundaries through digital services such as educational resource sharing platforms and telemedicine; in the organizational dimension, focusing on innovations in governmental governance and resource allocation, for example, linking public service expenditures to the degree of standardization of the relationship between the government and the business sector; in the environmental dimension, building a collaborative governance network of public services across administrative regions based on the strategy of regional economic integration. In the environmental dimension, relying on the regional economic integration strategy, a collaborative governance network of public services across administrative regions is constructed. This kind of systematic governance idea can fundamentally break down the systemic barriers of non-equalization of urban and rural public services, provide practical inspiration for the integrated development of urban and rural areas in the new era, and provide practical paths for realizing the goal of common prosperity.

#### 5.3.1 Implications for technology policy

The three types of high urban–rural income disparity groupings identified in the study provide a grouping tool for “problem diagnosis” in the precise design of policies. For example, for Xuzhou in northern Jiangsu Province and other areas with “insufficient public services and digital infrastructure,” we can increase investment in rural digital infrastructure (e.g., 5G networks, e-commerce platforms) and set up a mechanism for cross-regional collaboration in the provision of public services in the Yangtze River Delta; for Chuzhou in Anhui Province and other cities with “a lack of factor resources,” we need to enhance economic capacity through industrial cooperation parks and set up a mechanism for the provision of public services across the Yangtze River Delta. For “total factor resource-poor” cities such as Chuzhou in Anhui Province, it is necessary to enhance the economic capacity through industrial cooperation parks and at the same time, set up a special fund for fiscal transfers to clarify the rigid proportion of rural public service expenditures. This kind of policy intervention based on group characteristics can break through the traditional “one-size-fits-all” policy model and realize the precision and efficiency of resource allocation.

#### 5.3.2 Implications for fiscal governance

The empirical analysis of the Yangtze River Delta Urban Agglomeration shows that the formation of non-high urban–rural income disparity relies on multiple paths, such as technological, organizational, and environmental conditions. This conclusion provides differentiated references for regions at different stages of development: economically developed regions (e.g., Shanghai Qingpu) can deepen the cross-regional fiscal synergy mechanism and incorporate digital infrastructure standards into the framework of urban–rural public service integration; manufacturing powerhouse cities (e.g., Suzhou) can optimize their fiscal expenditure structure and establish precise supply mechanisms such as digital service vouchers; and human capital-rich regions (e.g., Changzhou) need to strengthen the linkage between public services and vocational skills training, and enhance the efficiency of resource allocation through the human capital points system. This kind of policy adaptation based on regional grouping characteristics can effectively promote the deep coupling of urban–rural integration policies with local development conditions.

## 6 Limitations and future research

This study uses the TOE framework to investigate the urban–rural income difference in the Yangtze River Delta. However, due to limitations in research design and data availability, further research is needed in certain areas. The study focuses on typical scenarios of the YRD urban agglomeration. While it can provide insight into urban–rural integration and synergistic growth of regional institutions, the sample’s regional distinctiveness may limit the generalizability of the findings. For example, the YRD region generally has a high rate of financial self-sufficiency and a strong foundation of digital governance, whereas less developed regions in the central and western regions may be more constrained by a lack of financial capacity and the rigidity of the household registration system (58), and the driving patterns and inhibiting paths of the urban–rural gap may show significant differences. Future research could expand to regional comparisons across economic gradients to reveal the differentiating effects of public service patterns at different stages of development.

Second, the study uses cross-sectional data to characterize static group relationships, which does not capture the dynamic lags of policy effects. For example, the “N-curve” effect of industrial structure upgrading on the urban–rural income gap (27) and the release of long-term dividends from the reform of the household registration system need to be tracked and observed through panel data. In the future, a more comprehensive analytical framework can be constructed by introducing a dynamic assessment model and combining it with policy text analysis.

Overall, this study provides a regional paradigm for the study of the configuration of the urban–rural income gap, and future studies can further reveal the spatial and temporal evolution of urban–rural basic public services on the urban–rural income gap through the expansion of samples and dynamic tracking in order to provide more generalized theoretical support for the precise design of urban–rural integration policies.

## Data Availability

The original contributions presented in the study are included in the article/supplementary material, further inquiries can be directed to the corresponding author.
